# The effects and safety of vasopressin receptor agonists in patients with septic shock: a meta-analysis and trial sequential analysis

**DOI:** 10.1186/s13054-019-2362-4

**Published:** 2019-03-14

**Authors:** Libing Jiang, Yi Sheng, Xia Feng, Jing Wu

**Affiliations:** 1 Yuhang Branch of The Second Affiliated Hospital of Zhenjiang University, No.369 Yingbin Road, Yuhang District, Hangzhou, 311100 Zhenjiang Province China; 2 Department of Emergency Medicine, Second Affiliated Hospital, School of Medicine & Institute of Emergency Medicine, Zhejiang University, No 88, Jiefang Rd, Hangzhou, China; 3 Department of Respiratory, The Third People’s Hospital of Hangzhou, West Lake Avenue 38, Hangzhou, China

**Keywords:** Vasopressin, Catecholamine, Septic shock, Meta-analysis

## Abstract

**Background:**

The aim of this study was to evaluate the effects and safety of vasopressin receptor agonists in patients with septic shock.

**Methods:**

PubMed, EMBASE, and Cochrane library were searched for randomized controlled trials evaluating the effects of vasopressin receptor agonists in septic shock patients. Two reviewers performed literature selection, data extraction, and quality evaluation independently. The primary outcome was mortality. And secondary outcomes included intensive care unit (ICU) length of stay, duration of mechanical ventilation, and incidence of adverse events. In addition, a trial sequential analysis (TSA) was performed.

**Results:**

Twenty studies were eligible for meta-analysis. The results showed vasopressin receptor agonists use was associated with reduced mortality (relative risk (RR) 0.92; 95% confidence interval (CI) 0.84 to 0.99; *I*^2^ = 0%). Nevertheless, they had no significant effects on ICU length of stay (mean deviation (MD) − 0.08, 95% CI, − 0.68 to 0.52, *I*^2^ = 0%) and duration of mechanical ventilation (MD − 0.58, 95% CI − 1.47 to 0.31, *I*^2^ = 57%). Additionally, there was no significant difference in total adverse events between two groups (RR 1.28, 95% CI 0.87 to 1.90, *I*^2^ = 57%), but vasopressin receptor agonists administration could significantly increase the risk of digital ischemia (RR 4.85, 95% CI 2.81 to 8.39, *I*^2^ = 26%). Finally, there was no statistical difference of cardiovascular events (RR 0.91, 95% CI 0.53 to 1.57, *I*^2^ = 1%), arrhythmia (0.77, 95% CI 0.48 to 1.23, *I*^2^ = 23%), mesenteric ischemia (0.83, 95% CI 0.44 to 1.55, *I*^2^ = 0%), diarrhea (2.47, 95% CI 0.77 to 7.96, *I*^2^ = 49%), cerebrovascular events (1.36, 95% CI 0.18 to 10.54, *I*^2^ = 0%), and hyponatremia (1.47, 95% CI 0.84 to 2.55, *I*^2^ = 0%) between two groups. Egger’s test showed there was no significant publication bias among studies (*P* = 0.36).

**Conclusions:**

The use of vasopressin might result in reduced mortality in patients with septic shock. An increased risk of digital ischemia must be taken into account.

**Electronic supplementary material:**

The online version of this article (10.1186/s13054-019-2362-4) contains supplementary material, which is available to authorized users.

## Background

Septic shock is the leading cause of death in intensive care units. It is reported that the mortality rate of these patients can be as high as 30–60% [1–3]. Maintaining effective blood pressure is important for these patients [4]. Vasopressors are often used to reach a target mean arterial pressure (MAP), after adequate fluid resuscitation. Catecholamines, such as norepinephrine (NE), are still the first-line drugs. However, high dose of catecholamines may be associated with a higher risk of complications, including myocardial ischemia, decreased cardiac output, arrhythmias, increased tissue oxygen consumption, and pulmonary hypertension [4, 5].

Relative vasopressin deficiency often occurs in septic shock patients [6, 7]. Some pre-clinical studies showed exogenous administration of vasopressin could increase the vascular tone and improve blood pressure [8]. Several clinical studies also reported early concomitant vasopressin, and norepinephrine therapy could reduce the dose of NE, shorten the time of achieving target mean arterial pressure, and reduce catecholamine-related complications [9, 10]. Therefore, the newest Surviving Sepsis guideline suggests vasopressin could be used to raise blood pressure to target mean arterial pressure or decrease norepinephrine dosage with weak recommendations [11]. However, no consensus has been made regarding the effects of vasopressin receptor agonists on patient-centered outcomes, especially mortality. The aim of this study is to explore the effects and safety of vasopressin receptor agonists in patients with septic shock.

## Methods

The present meta-analysis was performed and reported according to the Preferred Reporting Items for Systematic Reviews and Meta-Analyses (PRISMA, http://www.prisma-statement.org/).

### Registration and protocol

This meta-analysis was registered on PROSPERO (CRD42018104027).

### Inclusion criteria

Patients: Adult septic shock patients

Intervention: Vasopressin or its analogues (e.g., terlipressin, selepressin) with or without concomitant catecholamines, irrespective of dose and duration

Comparison: Catecholamines use alone, irrespective of dose and duration

Outcomes: The primary endpoint was 28/30-day mortality, and hospital mortality and ICU mortality were equal for this analysis module. The secondary endpoints included ICU length of stay, duration of mechanical ventilation, and adverse events (total adverse events, digital ischemia, cardiovascular events, arrhythmia, mesenteric ischemia, diarrhea, cerebrovascular events, and hyponatremia).

### Data source and literature search

PubMed, EMBASE, and Cochrane library were searched from inception to July 31, 2018. The detailed search strategy is showed in Additional file 1: Table S1. The bibliography of relevant articles was searched for additional articles. In addition, https://clinicaltrials.gov/ was searched for ongoing or unpublished studies.

### Study selection and data extraction

Two reviewers performed literature selection independently. Firstly, we excluded duplicates through reference management tool. Then, we exclude clearly non-relevant articles by reading titles and abstracts. Finally, we decided the eligibility of each article by full-text reading.

The same two reviewers did the data extraction independently using a pre-defined datasheet. And we recorded basic information of each eligible study, characteristics of included patients, interventions, comparisons, endpoints, and other items which were essential for quality evaluation. Any discrepancy was solved by discussion or consulting with the third reviewer.

### Study quality evaluation

Two reviewers evaluated the quality of each included study based on the following domains: sequence generation, allocation concealment, blinding of patients and personnel, blinding of outcome assessors, incomplete outcome data, and selective reporting. Each domain is classified as low risk, unclear risk, and high risk. Any discrepancy was solved by discussion or consulting with the third reviewer.

### Statistical analysis

Relative risk (RR) was used for dichotomous data, and mean difference (MD) was used for continuous data. The heterogeneity between studies was assessed using the *I*^2^ test and chi-square test. *P* < 0.1 and *I*^2^ ≥ 50% indicated significant heterogeneity, and the random effects model was used. Otherwise, the fixed effects model was used. A two-sided *P* value < 0.05 was considered statistically significant. Publication bias was assessed by funnel plot and Egger’s test quantitatively. All statistical analyses were performed using STATA 12.0 software (SERIAL NO.40120519635) and RevMan 5.3 (The Nordic Cochrane Centre, The Cochrane Collaboration, Copenhagen, Denmark).

In the present study, we used the GRADE (Grades of Recommendation, Assessment, Development, and Evaluation) to evaluate the quality of evidence. And evidences were categorized as high, moderate, low, and very low, according to two group factors (factors that can reduce the quality of the evidence and factors that can increase the quality of the evidence). This process was performed on GRADEpro GDT (https://gradepro.org/).

In the present study, we performed the trial sequential analysis (TSA) to decrease the risks of random errors due to sparse data and repetitive testing and calculate the optimal information size for this meta-analysis. In addition to the optimal information size, an adjusted boundary line for favoring vasopressin or its analogue use and an adjusted boundary line for favoring catecholamine use alone were generated to decide whether the meta-analysis should be terminated early or the confidence interval. In this TSA model, type I error was set at 5% and type II error was set at 20%. A 10% relative risk reduction (RRR) and baseline mortality calculated from the actual meta-analyses were used to calculate the optimal information size. TSA was performed using the trial sequential analysis v.0.9.5.10 beta (Copenhagen Trial Unit, Centre for Clinical Intervention Research, Rigshospitalet, Copenhagen, Denmark, available from www.ctu.dk/tsa).

## Results

### Literature selection process

**Fig. 1:**
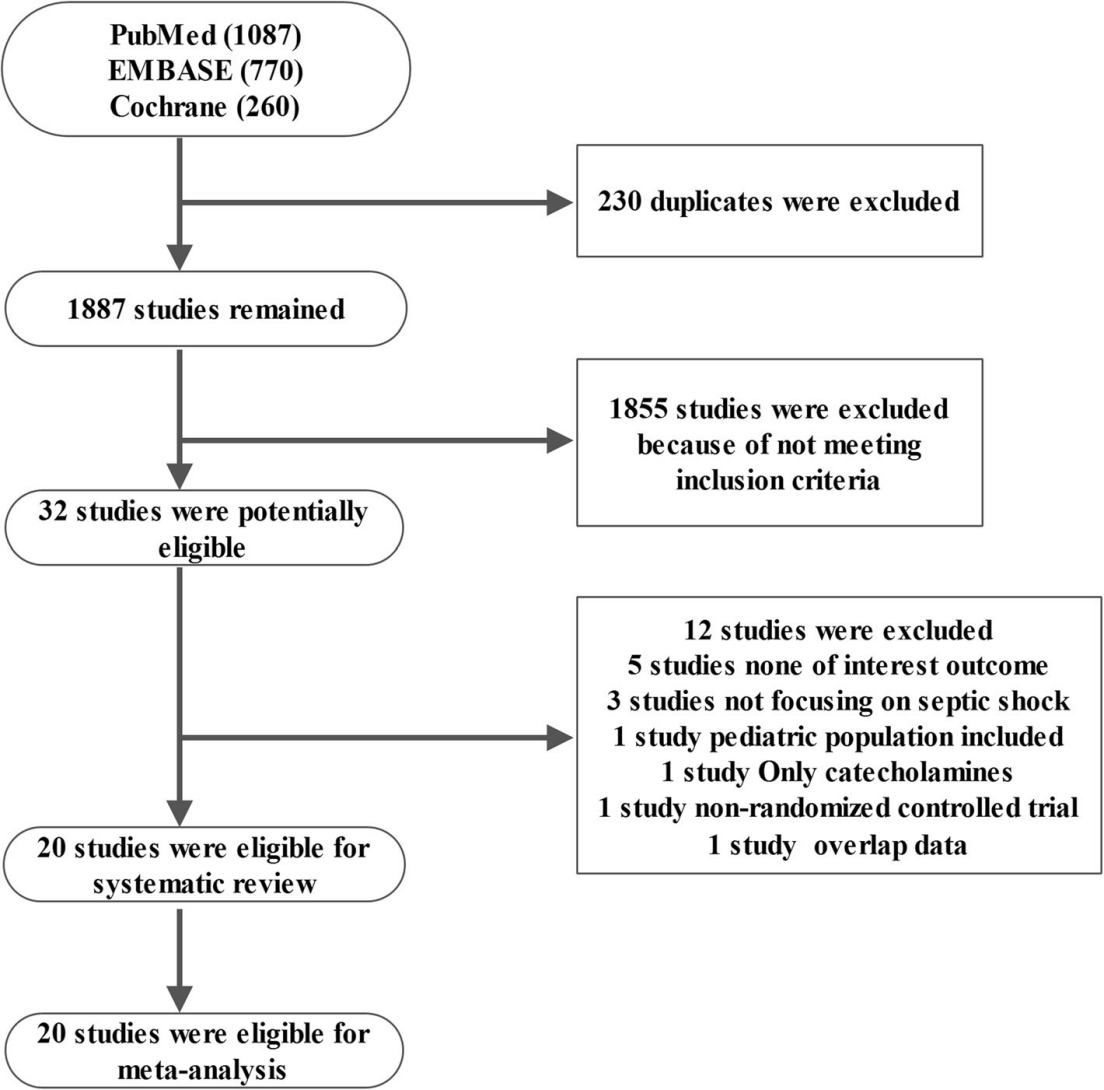
Flow chart of literature selection

After excluding 230 duplicates, 1887 studies from 2117 hits were chosen for further evaluation. Through reading title and abstract, 1855 studies were excluded and 32 studies were potentially eligible for evaluation by reading the full text. Finally, 20 studies were included for meta-analysis [12–31]. Figure 1 shows the process of literature selection and reasons for study exclusion. Detailed information of excluded studies and ongoing studies is presented in Additional file 1: Table S2, S3.

### The characteristics and quality of the included studies

Twenty studies [12–31] with 2250 septic shock patients who received vasopressin receptor agonists and 2281 septic shock patients who received catecholamine alone were eligible. Patients in 9 studies and 11 studies received vasopressin [12, 17, 19–21, 28–31] and vasopressin’s analogues (pituitrin 1 [14], selepressin 1 [23], terlipressin 9 [13, 15, 16, 18, 22, 24–27]), respectively. Among them, four studies [12, 17, 18, 21] were published in abstract and relevant data were obtained from the study by McIntyre et al. [32]. Detailed information is showed in Table 1, and quality evaluation of all included studies is showed in Additional file 1: Figures S1, S2.

**Table 1:** The characteristics of included studies

Study	Year	No. of patients	Patients	Intervention	Comparison	Outcome
Malay	1999	10	Septic shock	Vasopressin 0.04 U/min	NE	24 h
Albanese	2005	20	Septic shock	Terlipressin: one bolus of 1 mg and a second bolus of 1 mg was given if the MAP < 65 mmHg after 20 mins	NE was started at a dose of 0.3 μg/kg/min, followed by 0.3 μg/kg/min increments at 4-min intervals to raise MAP to 65 to 75 mmHg	Hospital
Lauzier	2006	23	Septic shock	Arginine-vasopressin 0.04–0.20 U/min	NE 0.1–2.8 μg/kg/min	ICU
Russell	2008	799	Septic shock	Vasopressin 0.01–0.03 U/min or at clinician’s discretion	NE 5 to 15 mg/min or at clinician’s discretion	90 days
Acevedo	2009	24	Septic shock and cirrhosis	Terlipressin 1–2 mg/4 h plus alpha-adrenergic drugs	Alpha-adrenergic drugs alone	Hospital
Morelli	2009	45	Septic shock	Terlipressin 1.3 μg/kg/h Vasopressin 0.03 U/min	NE 15 μg/min	ICU
Han	2012	139	Septic shock	Pituitrin 1.0–2.5 U/h	Dopamine or NE 2–20 μg/kg/h	28 days
Svoboda	2012	30	Septic shock	Terlipressin 4 mg/24 h for 72 h plus open-label norepinephrine	NE > 0.6 μg/kg/min for more than 24 h	28 days
Fonseca Ruiz	2013	30	Septic shock	Vasopressin 0.01–0.04 U/min plus NE	NE	28 days
Hua	2013	32	Septic shock patients with ARDS	Terlipressin 1.3 mg/kg/h	Dopamine < 20 mg/kg/min	28 days
Oliveira	2014	387	Septic shock	Vasopressin 0.01–0.03 U/min with low doses of norepinephrine	NE 0.05–2.0 μg/kg/min	28 days
Barzegar	2016	30	Septic shock	Vasopressin 0.03 μg/min plus NE	NE: infusion adjusted to MAP ≥ 65 mmHg	28 days
Choudhury	2016	84	Cirrhotics with septic shock	Terlipressin 2–8 mg over 24 h	NE 7.5–60 μg/min	28 days
Clem	2016	82	Septic shock	Vasopressin 0.04 U/min plus NE with 0.05–0.5 μg/kg/min	NE 0.05 to 0.5 μg/kg/min	28 days
Gordon	2016	408	Septic shock	Vasopressin: titrated up to 0.06 U/min to maintain the MAP 65 to 75 mmHg	NE: titrated up to 12 μg/min to maintain the MAP 65 to 75 mmHg	28 days
Capoletto	2017	250	Septic shock and cancer	Vasopressin	NE	90 days
Chen	2017	57	Septic shock patients with ARDS	Terlipressin 0.01–0.04 U/min to maintain MAP between 65 and 75 mmHg, if necessary plus NE	NE > 1 μg/min to maintain MAP between 65 and 75 mmHg	28 days
Prakash	2017	184	Cirrhosis with septic shock.	Terlipressin 2 mg/24 h and 3.75–30 μg/min of NE as needed to maintain MAP > 65 mmHg	NE 7.5–60 μg/min	30 days
Russell	2017	48	Septic shock	Selepressin 1.25, 2.5, and 3.75 ng/kg/min until shock resolution or a maximum of 7 days	Placebo	28 days
Liu	2018	535	Septic shock	Terlipressin 20–160 μg/h	NE 4–30 μg/min	28 days

*No* number, *NE* norepinephrine, *ICU* intensive care unit, *ARDS* acute respiratory distress syndrome

### Meta-analysis

#### The primary endpoint: mortality

**Fig. 2:**
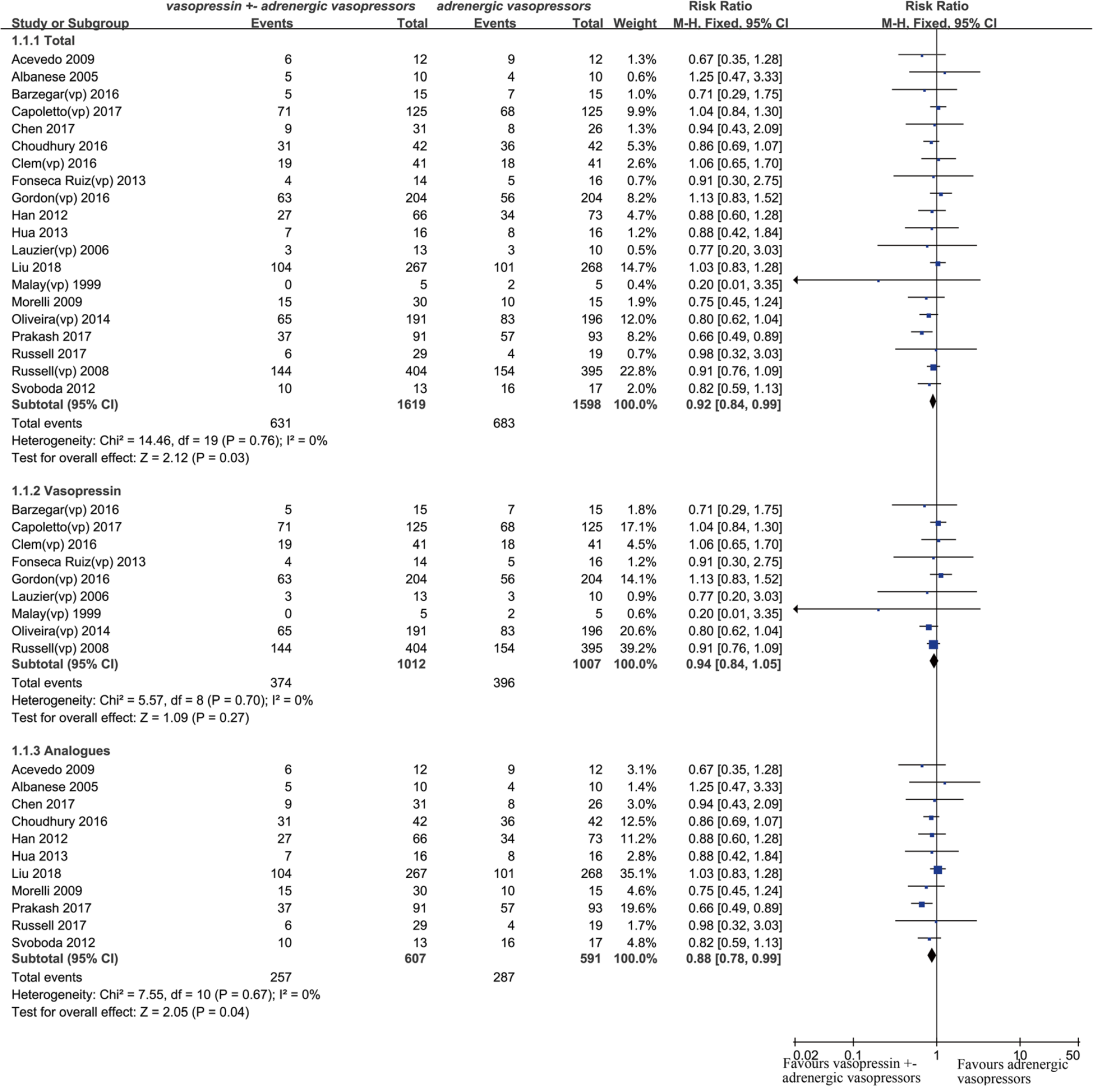
Forest plot for effects of vasopressin or its analogues on 28/30-day mortality (mortality rate within 30 was equal)

**Fig. 3:**
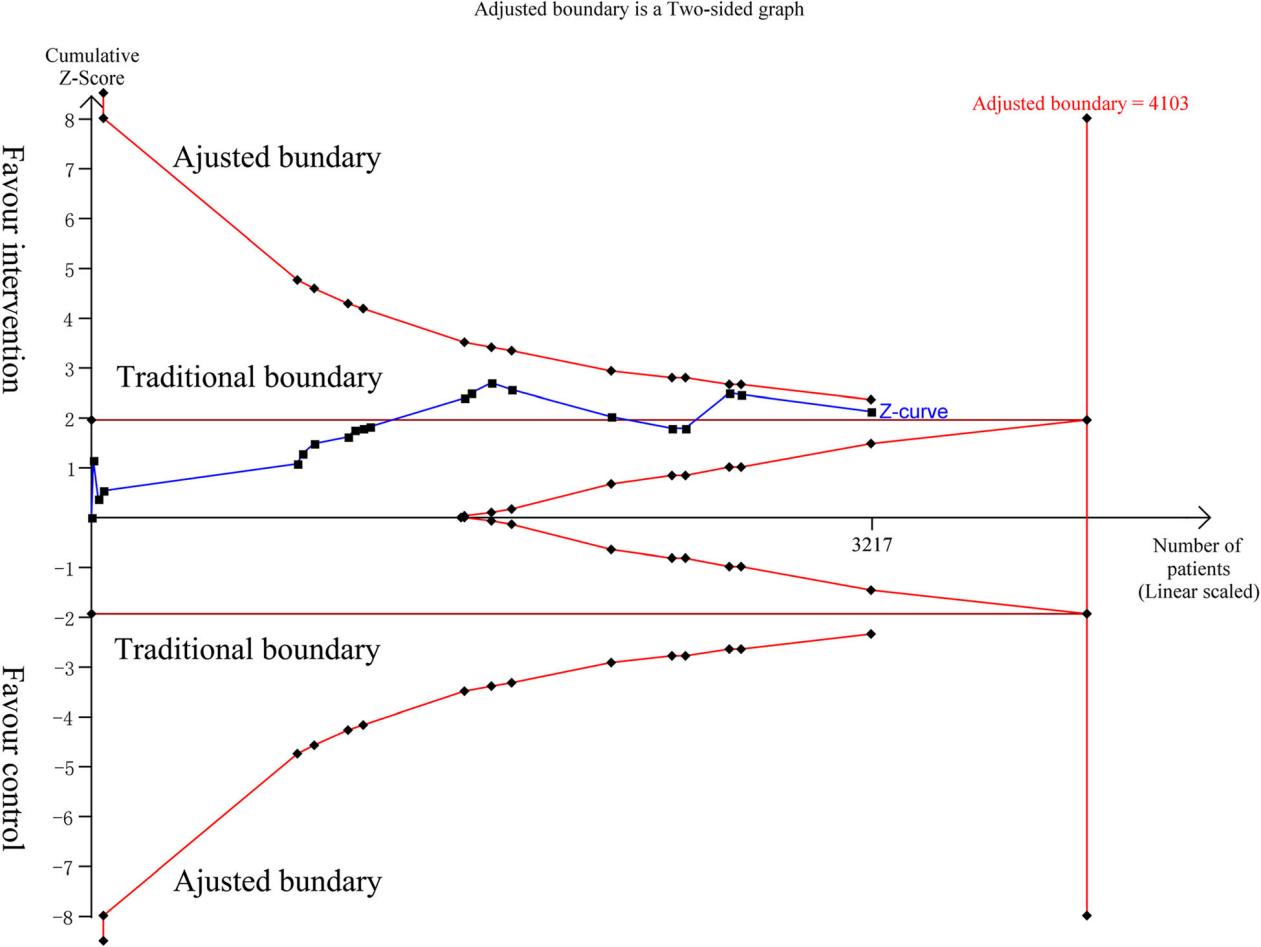
Trial sequential analysis for effects of vasopressin or its analogues on 28/30-day mortality. The diversity-adjusted required information size (4103 participants) was based on a relative risk reduction of 10%, an alpha of 5%, a beta of 20%, and an event proportion of 43% in the control arm. The blue cumulative *z* curve was constructed using a fixed effects model

Twenty studies were included for mortality analysis [12–31], and the combined RR was 0.92 (95% confidence interval (CI) 0.84 to 0.99, *P* = 0.03, *I*^2^ = 0%) (Fig. 2). The quality of evidence is presented in Additional file 1: Table S4. The results of TSA indicated the optimal information size was 4103 patients for mortality and more high-quality RCTs are needed, although *z* curve had crossed the general boundary line, but it did not cross any adjusted boundary line favoring the intervention group or control group. And the adjusted RR was 0.92 (95% CI 0.84 to 0.99, *P* = 0.03, *I*^2^ = 0%), based on 10% RRR (from a baseline event rate of 43%) (Fig. 3).

#### Post hoc sensitive and subgroup analysis

Firstly, the combined RR was 0.95 (95% CI 0.86–1.05, *P* = 0.30, *I*^2^ = 0%) [13–16, 19, 20, 22–31] for studies published in full text and 0.85 (95% CI 0.74–0.98, *P* = 0.02, *I*^2^ = 23%) [12, 17, 18, 21] for studies published in abstract. In addition, after removing three studies that did not report 28/30-day mortality, the combined RR was 0.92 (95% CI 0.83–1.00, *P* = 0.06, *I*^2^ = 0%) [12–15, 17–19, 21–29, 31]. Thirdly, the combined RR was 0.94 (95% CI 0.84–1.05, *P* = 0.70, *I*^2^ = 0%) [12, 17, 19–21, 28–31] for patients who received vasopressin and 0.88 (95% CI 0.78–0.99, *P* = 0.04, *I*^2^ = 0%) [13–16, 18, 22–27] for patients who received its analogues (Fig. 2). Finally, we performed another subgroup analysis based on different diagnoses. In patients with cirrhosis, the combined RR was 0.73 (95% CI 0.61–0.88, *P* = 0.001, *I*^2^ = 23%), and in other patients, the combined RR was 0.95 (95% CI 0.87–1.04, *P* = 0.24, *I*^2^ = 0%).

### The secondary endpoints

#### ICU length of stay

**Fig. 4:**
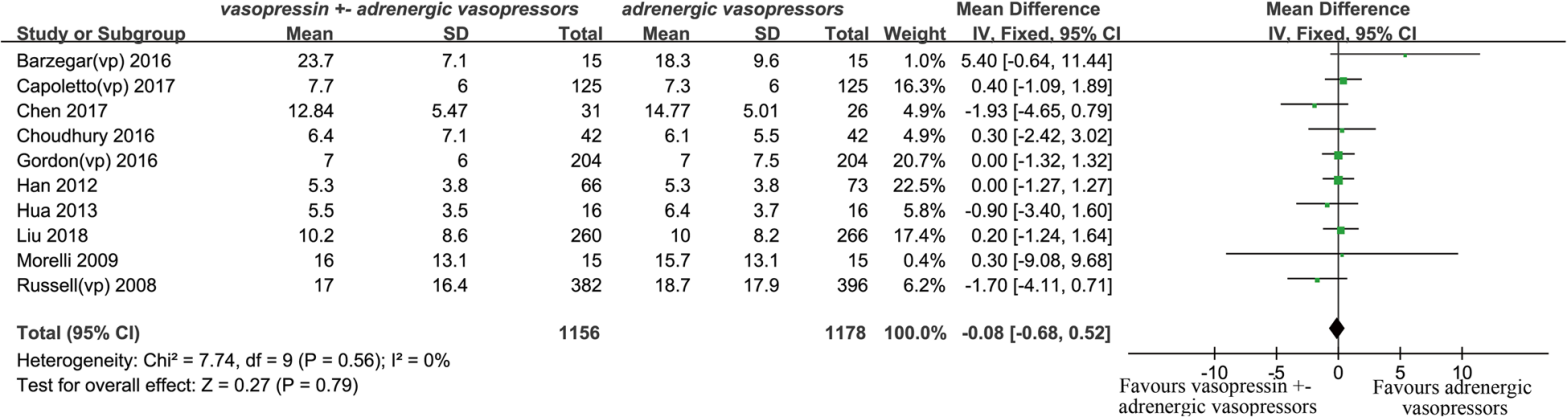
Forest plot for effects of vasopressin or its analogues on intensive care unit length of stay

Ten studies reported ICU length of stay [12, 14–16, 19, 22, 24, 27, 28, 31]. The results showed there were no effects of vasopressin receptor agonists on ICU length of stay (MD − 0.08, 95% CI − 0.68–0.52, *P* = 0.79, *I*^2^ = 0%) (Fig. 4).

#### Duration of mechanical ventilation

**Fig. 5:**
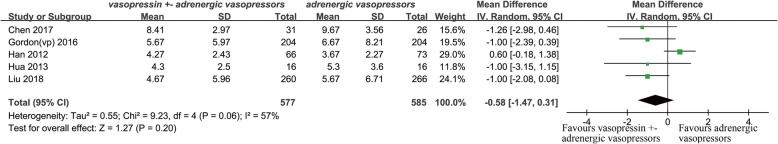
Forest plot for effects of vasopressin or its analogues on the duration of mechanical ventilation

Five studies were eligible for analysis of duration of mechanical ventilation [14, 15, 19, 24, 27]. The combined MD was − 0.58 (95% CI − 1.47–0.31, *P* = 0.20, *I*^2^ = 57%) (Fig. 5). The results showed vasopressin receptor agonist administration did not significantly affect the duration of mechanical ventilation. The result of the study by Han was different from the other studies [14]. A sensitive analysis was performed by removing the study by Han; the combined MD was − 1.05 (95% CI − 1.77 to − 0.32, *P* = 0.005, *I*^2^ = 0%).

### Adverse events

#### Total adverse events

**Fig. 6:**
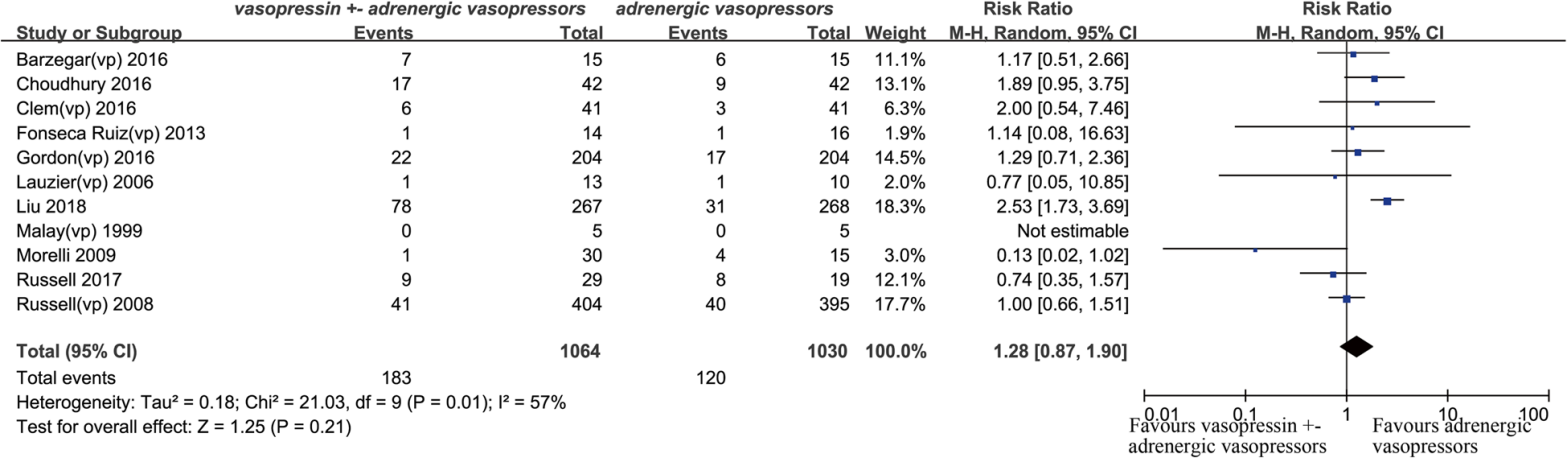
Forest plot for effects of vasopressin or its analogues on total adverse events

Eleven studies were included in the analysis of total adverse events [16, 19–23, 27–31]. The combined RR was 1.28 (95% CI 0.87–1.90, *P* = 0.21, *I*^2^ = 57%) (Fig. 6). The results were driven by the study by Liu et al. [27], which carried 18.3% of the weight. A sensitive analysis was performed by removing the study by Liu et al., and the combined RR was 1.11 (95% CI 0.86–1.43, *P* = 0.44, *I*^2^ = 10%).

#### Digital ischemia

**Fig. 7:**
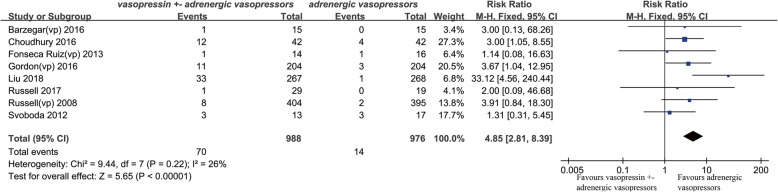
Forest plot for effects of vasopressin or its analogues on digital ischemia

Eight studies and 1964 patients were eligible for the analysis [19, 22, 23, 25, 27–29, 31]. The combined RR was 4.85 (95% CI 2.81–8.39, *P* < 0.001, *I*^2^ = 26%) (Fig. 7), indicating that the use of vasopressin receptor agonists was associated with more digital ischemia events. A sensitive analysis was performed by removing the study by Liu et al. [27], due to its results that were significantly different from the other studies. And the combined RR was 2.79 (95% CI 1.54–5.05, *P* < 0.001, *I*^2^ = 0%), supporting the original conclusion.

#### Other adverse events

There were no effects of vasopressin receptor agonists on cardiovascular events [19, 23, 27, 28, 30, 31], arrhythmia [16, 19, 21–23, 27, 28, 31], mesenteric ischemia [19, 23, 27, 31], diarrhea [23, 27, 31], cerebrovascular events [23, 31], and hyponatremia [27, 28, 31]. And the combined RR was 0.91 (95% CI 0.53–1.57, *P* = 0.73, *I*^2^ = 1%) (Additional file 1: Figure S3), 0.77 (95% CI 0.48–1.23, *P* = 0.28, *I*^2^ = 23%) (Additional file 1: Figure S4), 0.83 (95% CI 0.44–1.55, *P* = 0.55, *I*^2^ = 0%) (Additional file 1: Figure S5), 2.47 (95% CI 0.77–7.96, *P* = 0.13, *I*^2^ = 49%) (Additional file 1: Figure S6), 1.36 (95% CI 0.18–10.54, *P* = 0.77, *I*^2^ = 0%) (Additional file 1: Figure S7), and 1.47 (95% CI 0.84–2.55, *P* = 0.18, *I*^2^ = 0%) (Additional file 1: Figure S8), respectively. Additional subgroup analyses are showed in Table 2.

**Table 2:** Subgroup analysis based on medication type

Indicator	Vasopressin	Vasopressin’s analogues
ICU length of stay (MD)	− 0.17 (95% CI − 0.98–0.63, *P* = 0.67, *I*^2^ = 0%)	0.03 (95% CI − 0.87–0.93, *P* = 0.94, *I*^2^ = 24%)
Duration of MV (MD)	− 1.00 (95% CI −2.39-0.39, *P* = 0.16)*	− 0.50 (95% CI − 1.57–0.57, *P* = 0.36, *I*^2^ = 63%)
Total adverse events (RR)	1.13 (95% CI 0.83–1.53, *P* = 0.43, *I*^2^ = 0%)	1.20 (95% CI 0.52–2.74, *P* = 0.67, *I*^2^ = 79%)
Digital ischemia (RR)	3.33 (95% CI 1.39–7.95, *P* < 0.001, *I*^2^ = 0%)	6.06 (95% CI 2.97–12.37, *P* < 0.001, *I*^2^ = 68%)
Cardiovascular events (RR)	0.93 (95% CI 0.51–1.69, *P* = 0.80, *I*^2^ = 27%)	0.84 (95% CI 0.24–2.99, *P* = 0.79, *I*^2^ = 0%)
Arrhythmia (RR)	0.99 (95% CI 0.51–1.91, *P* = 0.98, *I*^2^ = 15%)	0.57 (95% CI 0.29–1.15, *P* = 0.12, *I*^2^ = 35%)
Mesenteric ischemia (RR)	0.77 (95% CI 0.38–1.53, *P* = 0.45, *I*^2^ = 0%)	1.22 (95% CI 0.26–5.64, *P* = 0.80, *I*^2^ = 42%)
Diarrhea (RR)	0.98 (95% CI 0.06–15.58)*	1.64 (95% CI 0.05–54.19, *P* = 0.78, I^2^ = 71%)
Cerebrovascular events (RR)	0.98 (95% CI 0.06–15.58, *P* = 0.99)*	2.00 (95% CI 0.09–46.68, *P* = 0.67)*
Hyponatremia (RR)	2.31 (95% CI 0.35–15.09, *P* = 0.38, *I*^2^ = 0%)	1.39 (95% CI 0.78–2.49, *P* = 0.26)*

*RR* relative risk, *MD* mean difference, *CI* confidence interval, *ICU* intensive care unit, *MV* mechanical ventilation

*Only one study

#### Publication bias

Publication bias was assessed via funnel plots and Egger’s test (Additional file 1: Figure S9). The results of Egger’s test indicated there was no significant publication bias among the included studies (*P* = 0.39).

## Discussion

In this meta-analysis, the authors evaluated the effects and safety of vasopressin receptor agonists in patients with septic shock. The results showed vasopressin receptor agonist administration might be associated with increased survival in septic shock patients and further studies are required. However, their use could increase the risk of digital ischemia. There were no effects on ICU length of stay, duration of mechanical ventilation, cardiovascular ischemia events, arrhythmia, cerebrovascular ischemia events, mesenteric ischemia, diarrhea, and hypomania.

Generally, catecholamines, especially norepinephrine, are used as the first-line vasopressors in septic shock patients [3, 33–35]. However, with a better understanding of the pathophysiology of septic shock and growing attention to the side effects of catecholamines, alternative vasopressors are searched. Vasopressin is an endogenous hormone, and the supraoptic and paraventricular hypothalamic nuclei are the principal sources [36, 37]. Plasma vasopressin level in normal subjects does not exceed 4 pg/ml. But in patients with septic shock, the level of plasma vasopressin is reported to be abnormally low [36–39]. Moreover, exogenously administered vasopressin could increase the responsiveness to infused catecholamines and reduce the dose of catecholamines [40–42].

Vasopressin in Septic Shock Trial (VASST) failed to find a statistical difference in short-term and long-term mortality between septic shock patients who received vasopressin and norepinephrine [31]. In the present meta-analysis, we find the use of vasopressin receptor agonists is associated with increased survival when compared with those that received catecholamines alone, and this positive association may be more obvious in patients with cirrhosis who received terlipressin. Terlipressin, a synthetic analogue of vasopressin with a longer half-life, acts via V1 receptors on arteriolar smooth muscle cells. Terlipressin is generally used for hepatorenal syndrome and esophageal variceal bleeding [43, 44]. Previous small studies found a continuous infusion of terlipressin might be more effective than vasopressin in restoring hemodynamic status with less adverse events [16, 25, 45]. In the study by Choudhury et al. [22], the authors even found terlipressin is effective in improving survival of cirrhotics with septic shock, and they suggested early introduction of terlipressin rather than after failure of monotherapy. This is in agreement with the results of our study. The survival advantage of terlipressin is more obvious in cirrhotics with septic shock perhaps because it can reduce the portal pressure and result in redistribution of splanchnic blood. Additionally, terlipressin use may be useful in renal function recovery [22]. Selepressin, a more selective V1a receptor agonist, was reported to be effective in the improvement of hemodynamics in septic shock animal models and decreasing pulmonary capillary leak when used early or as first-line agent [46–48]. One small phase IIb human study reported selepressin was safe and effective in septic shock patients [49].

Several meta-analyses reached conflict conclusions [32, 50–54]. Possible reasons include different inclusion criteria. In this present study, both studies in full text and abstract were eligible. In order to reduce patient heterogeneity, only septic shock patients were included in the present study. Additionally, different endpoints and statistical methods may also account for the inconsistent outcomes.

### The limitation of this study

Several limitations of the present study should be concerned. Firstly, although there was no statistical significance of Egger’s test, the possibility of publication bias cannot be completely excluded. Secondly, some endpoints were not reported in studies, which were published in the abstract. Thirdly, ICU mortality, 24 h mortality, hospital mortality, and 28/30-day mortality were regarded to be equal in the present study, and this might bias the outcome. Finally, long-term endpoints, like 90-day mortality, and some surrogate outcomes were not reported in the present study.

### The implication for clinical practice and further studies

The results of this meta-analysis showed vasopressin receptor agonists improved survival with a higher risk of digital ischemia. The following reasons may account for the higher incidence of digital ischemia in the study by Liu et al. Firstly, 94% of patients with digital ischemia in their study received terlipressin and open-label noradrenaline. Furthermore, the maximum dose of terlipressin used in their study was higher than that reported in other studies [27]. However, no patient needed surgical interventions for digital ischemia. Another concern of using vasopressin in patients with septic shock is its effects on cardiac output and oxygen delivery. Vasopressin has previously been reported to be associated with a reduction of cardiac output [55], although this association is not found in other studies [16, 56]. Factors including different infusion method and dose of vasopressin, different period of fluid resuscitation, and additional medication use (inotropic infusion) may partially explain the diverse results [56]. Neto et al., in their meta-analysis, pointed that vasopressin use did not result in decreased cardiac output, except for high dose of terlipressin [52]. Additionally, Gordon et al. and Neto et al., in their studies, found vasopressin administration was associated with a significant decrease in heart rate, and this may play important role in effect on the cardiac output of vasopressin [52, 56]. In most published studies, patients in the intervention group received both vasopressin and open-label catecholamines, and this may bias the outcome. And more head-to-head comparative randomized evidence is required. The VASST study found the survival advantage of concomitant vasopressin and norepinephrine therapy was obvious in patients with less severe shock [57]. In another study, lactate concentration was reported to be associated with the hemodynamic response of vasopressin [58]. In the study by Nascente et al., they found vasopressin administration is likely to improve microcirculation in septic shock patients whose baseline noradrenaline dose was higher than 0.38 μg/kg/min [59]. Therefore, uncovering specific subgroups of septic patients who are most likely to respond to early initiation of vasopressin is important [58]. A post hoc analysis pointed that the adjunctive use of corticosteroids could increase the survival benefit of vasopressin. And in these patients, the serum vasopressin concentration significantly increased [60]. Although this association did not been observed in the following randomized controlled trial [19], adjunctive treatments with vasopressin in septic shock patients are another point requiring more studies. Moreover, the best dose, time of use [10, 61–64], infusion method (continuous or intermittent), and discontinuation strategies are also a hot topic and remain unclear [65].

## Conclusions

The use of vasopressin might result in reduced mortality in patients with septic shock. An increased risk of digital ischemia must be taken into account, and more studies are required.

## Additional file

Additional file 1: **Table S1.** Study search strategy. **Table S2.** Information on excluded studies. **Table S3.** List of Ongoing studies. **Table S4.** GRADE. **Figure S1.** Risk of bias summary. **Figure S2.** Risk of bias graph. **Figure S3.** Forest plot for vasopressin or its analogues on cardiovascular events. **Figure S4.** Forest plot for vasopressin or its analogues on arrhythmia. **Figure S5.** Forest plot for vasopressin or its analogues on mesenteric ischemia events. **Figure S6.** Forest plot for vasopressin or its analogues on diarrhea. **Figure S7.** Forest plot for vasopressin or its analogues on cerebrovascular events. **Figure S8.** Forest plot for vasopressin or its analogues on hyponatremia. **Figure S7.** Funnel plot for publication bias. (DOCX 554 kb)

## Abbreviations

ARDS: Acute respiratory distress syndrome
CI: Confidence interval
ICU: Intensive care unit
MD: Mean difference
NE: Norepinephrine
RR: Relative risk
TSA: Trial sequential analysis
