# Supplementary material for: The effects and safety of vasopressin receptor agonists in patients with septic shock: a meta-analysis and trial sequential analysis
Source: Crit Care. 2019 Mar 14;23:91. doi: 10.1186/s13054-019-2362-4 (PMC6419432; doi:10.1186/s13054-019-2362-4)
Supplement: Supplementary file 1 — Table S1. Study search strategy. Table S2. Information on excluded studies. Table S3. List of Ongoing studies. Table S4. GRADE. Figure S1. Risk of bias summary. Figure S2. Risk of bias graph. Figure S3. Forest plot for vasopressin or its analogues on cardiovascular events. Figure S4. Forest plot for vasopressin or its analogues on arrhythmia. Figure S5. Forest plot for vasopressin or its analogues on mesenteric ischemia events. Figure S6. Forest plot for vasopressin or its analogues on diarrhea. Figure S7. Forest plot for vasopressin or its analogues on cerebrovascular events. Figure S8. Forest plot for vasopressin or its analogues on hyponatremia. Figure S7. Funnel plot for publication bias. (DOCX 554 kb) [file 13054_2019_2362_MOESM1_ESM.docx]

Catalogue

[Table S1 Stuty search strategy 1](#_Toc524727122)

[Table S2 Information on excluded studies 2](#_Toc524727123)

[Table S 3 List of Onging studies 3](#_Toc524727124)

[Table S4 GRADE 4](#_Toc524727125)

[Figure S1 Risk of bias summary 6](#_Toc524727126)

[Figure S2 Risk of bias graph 6](#_Toc524727127)

[Figure S3 Forest plot for vasopressin or its analogues on cardiovascular events. 7](#_Toc524727128)

[Figure S4 Forest plot for vasopressin or its analogues on arrhythmia 7](#_Toc524727129)

[Figure S5 Forest plot for vasopressin or its analogues on mesenteric ischemia events 7](#_Toc524727130)

[Figure S6 Forest plot for vasopressin or its analogues on diarrhea 7](#_Toc524727131)

[Figure S7 Forest plot for vasopressin or its analogues on cerebrovascular events 8](#_Toc524727132)

[Figure S8 Forest plot for vasopressin or its analogues on hyponatremia 8](#_Toc524727133)

[Figure S7 Funnel plot for publication bias 8](#_Toc524727134)

Table S1 Stuty search strategy

| **Database** | **Strategy** |
| --- | --- |
| PubMed | Search ((((("Shock, Septic"[Mesh]) OR "Sepsis"[Mesh]) OR (((((sepsi*[Title/Abstract]) OR septi*[Title/Abstract]) OR vasoplegic[Title/Abstract]) OR distributive[Title/Abstract]) OR vasodilatory[Title/Abstract]))) AND ((((((((("Vasopressins"[Mesh]) OR "Arginine Vasopressin"[Mesh]) OR "Deamino Arginine Vasopressin"[Mesh]) OR "Lypressin"[Mesh]) OR "Felypressin"[Mesh]) OR "Ornipressin"[Mesh]) OR "terlipressin"[Supplementary Concept])) OR (((((((((Vasopressin*[Title/Abstract]) OR Argipressin[Title/Abstract]) OR Desmopressin[Title/Abstract]) OR Lypressin[Title/Abstract]) OR Felypressin[Title/Abstract]) OR Ornipressin[Title/Abstract]) OR Terlipressin[Title/Abstract]) OR selepressin[Title/Abstract]) OR Glypressin[Title/Abstract]) OR Pituitrin[Title/Abstract]))) Filters: Publication date to 2018/07/31 |
| EMBASE | 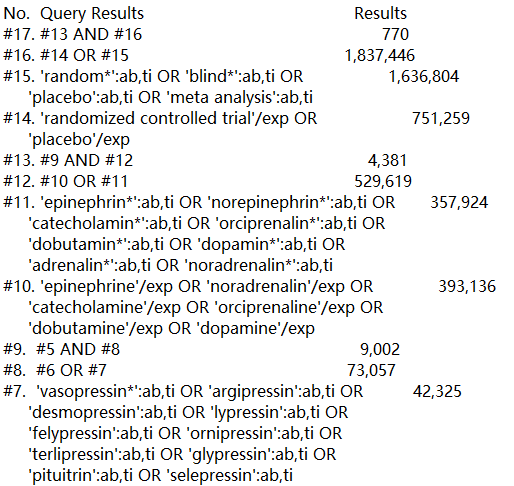  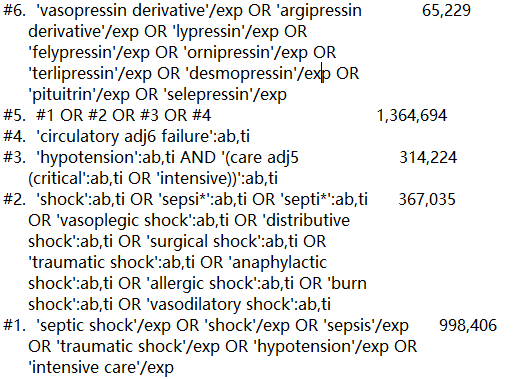 |
| Cochrane | 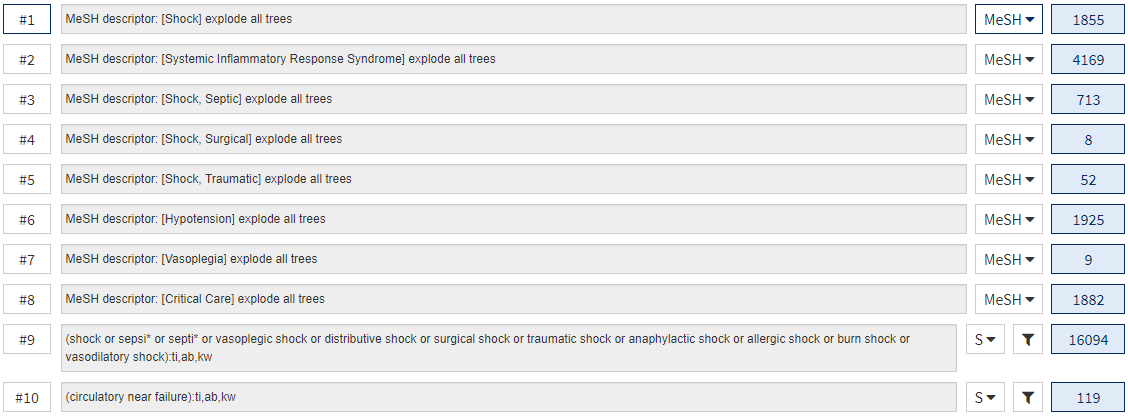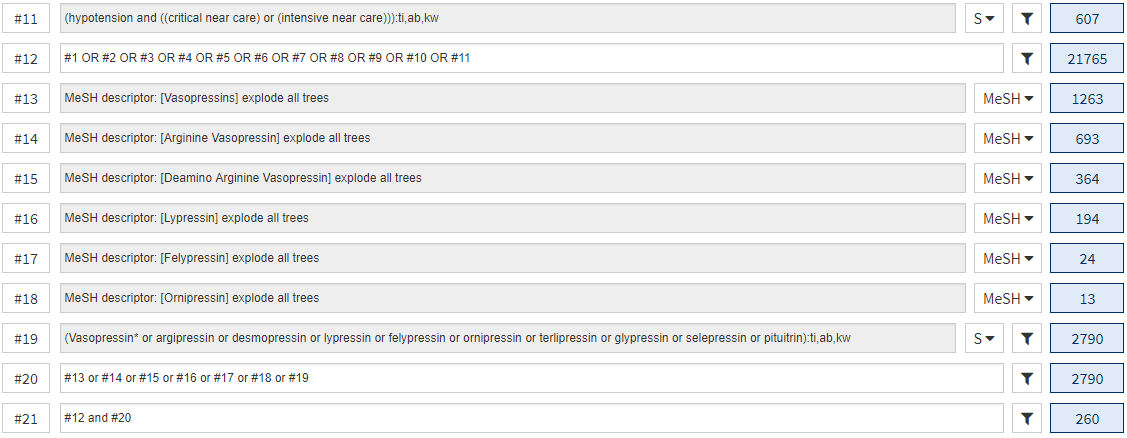 |

Table S2 Information on excluded studies

| Author | Year | Journal | Reason for exclusion |
| --- | --- | --- | --- |
| Patel MB [1] | 2002 | Anesthesiology | None of interest outcome |
| Dünser MW[2] | 2004 | Anesth Analg | Not focusing on septic shock |
| Dünser MW [3] | 2004 | Crit Care Med | Not focusing on septic shock |
| Mzezewa S [4] | 2004 | Burns | Pediatric population included |
| Morelli A [5] | 2008 | Crit Care | Only catecholamines |
| Elmenesy[6] | 2008 | Egyptian Journal of Anaesthesia. | None of interest outcome |
| Morelli A [7] | 2011 | Crit Care | None of interest outcome |
| Mukhtar A[8] | 2011 | Crit Care Med | Not focusing on septic shock |
| Barzegar[9] | 2017 | Indian J Crit Care Med | None of interest outcome |
| Nascente[10] | 2017 | Clinics | None of interest outcome |
| Hammond[11] | 2018 | Pharmacotherapy | Non-randomized controled trial |
| Barzegar[9] | 2017 | Indian J Crit Care Med | Overlaping data |

**Reference**

1. Patel BM, Chittock DR, Russell JA, Walley KR: **Beneficial effects of short-term vasopressin infusion during severe septic shock**. *Anesthesiology* 2002, **96**(3):576-582.

2. Dunser MW, Fries DR, Schobersberger W, Ulmer H, Wenzel V, Friesenecker B, Hasibeder WR, Mayr AJ: **Does arginine vasopressin influence the coagulation system in advanced vasodilatory shock with severe multiorgan dysfunction syndrome?** *Anesthesia and analgesia* 2004, **99**(1):201-206.

3. Dunser MW, Hasibeder WR, Wenzel V, Schwarz S, Ulmer H, Knotzer H, Pajk W, Friesenecker BE, Mayr AJ: **Endocrinologic response to vasopressin infusion in advanced vasodilatory shock**. *Critical care medicine* 2004, **32**(6):1266-1271.

4. Mzezewa S, Jonsson K, Aberg M, Sjoberg T, Salemark L: **A prospective double blind randomized study comparing the need for blood transfusion with terlipressin or a placebo during early excision and grafting of burns**. *Burns : journal of the International Society for Burn Injuries* 2004, **30**(3):236-240.

5. Morelli A, Ertmer C, Rehberg S, Lange M, Orecchioni A, Laderchi A, Bachetoni A, D'Alessandro M, Van Aken H, Pietropaoli P *et al*: **Phenylephrine versus norepinephrine for initial hemodynamic support of patients with septic shock: a randomized, controlled trial**. *Critical care (London, England)* 2008, **12**(6):R143.

6. Elmenesy TM, Nassar Y: **A randomized double-blind comparative study between short-term norepinephrine and vasopressin infusion in septic shock**. *Egyptian Journal of Anaesthesia* 2008, **24**(4):355-362.

7. Morelli A, Donati A, Ertmer C, Rehberg S, Kampmeier T, Orecchioni A, Di Russo A, D'Egidio A, Landoni G, Lombrano MR *et al*: **Effects of vasopressinergic receptor agonists on sublingual microcirculation in norepinephrine-dependent septic shock**. *Critical care (London, England)* 2011, **15**(5):R217.

8. Mukhtar A, Salah M, Aboulfetouh F, Obayah G, Samy M, Hassanien A, Bahaa M, Abdelaal A, Fathy M, Saeed H *et al*: **The use of terlipressin during living donor liver transplantation: Effects on systemic and splanchnic hemodynamics and renal function**. *Critical care medicine* 2011, **39**(6):1329-1334.

9. Barzegar E, Nouri M, Mousavi S, Ahmadi A, Mojtahedzadeh M: **Vasopressin in Septic Shock; Assessment of Sepsis Biomarkers: A Randomized, Controlled Trial**. *Indian journal of critical care medicine : peer-reviewed, official publication of Indian Society of Critical Care Medicine* 2017, **21**(9):578-584.

10. Nascente APM, Freitas FGR, Bakker J, Bafi AT, Ladeira RT, Azevedo LCP, Lima A, Machado FR: **Microcirculation improvement after short-term infusion of vasopressin in septic shock is dependent on noradrenaline**. *Clinics (Sao Paulo, Brazil)* 2017, **72**(12):750-757.

11. Hammond DA, Ficek OA, Painter JT, McCain K, Cullen J, Brotherton AL, Kakkera K, Chopra D, Meena N: **Prospective Open-label Trial of Early Concomitant Vasopressin and Norepinephrine Therapy versus Initial Norepinephrine Monotherapy in Septic Shock**. *Pharmacotherapy* 2018, **38**(5):531-538.

Table S 3 List of Onging studies

| **Title** | **Intervention** | **NCT number** |
| --- | --- | --- |
| Vasoactive Drugs in Intensive Care Unit | Drug: Norepinephrine  Drug: Epinephrine  Drug: Phenylephrine  Drug: Vasopressin | NCT02118467 |
| Evaluation of Early Association of Terlipressin and Norepinephrine During Septic Shock; the TerliNor Study (TERLINOR) | Drug: Terlipressin associated with norepinephrine  Drug: Placebo (physiologic serum) associated with norepinephrine | NCT03336814 |
| Terlipressin Alone Versus the Standard Therapy With Catecholamines for Hepatic Patients With Septic Shock- Prospective Single Center Randomized Controlled Study | Drug:Terlipressin alone  Drug: the standard therapy (norepinephrine +/- epinephrine) | NCT03608514 |
| What Should be the Next Vasopressor for Severe Septic Shock? Methylene Blue or Terlipressin | Drug: Methylene Blue  Drug: Terlipressin | NCT03038503 |

Table S4 GRADE

| **Certainty assessment** | | | | | | | **Summary of findings** | | | | |
| --- | --- | --- | --- | --- | --- | --- | --- | --- | --- | --- | --- |
| **№ of participants (studies) Follow-up** | **Risk of bias** | **Inconsistency** | **Indirectness** | **Imprecision** | **Publication bias** | **Overall certainty of evidence** | **Study event rates (%)** | | **Relative effect (95% CI)** | **Anticipated absolute effects** | |
|  |  |  |  |  |  |  | **With placebo** | **With Primary endpoints** |  | **Risk with placebo** | **Risk difference with Primary endpoints** |
| **28/30 days mortality** | | | | | | | | | | | |
| 3217 (20 RCTs) | serious ^a^ | not serious | not serious | not serious | none | ⨁⨁⨁◯ MODERATE | 683/1598 (42.7%) | 631/1619 (39.0%) | **RR 0.92** (0.84 to 0.99) | 427 per 1,000 | **34 fewer per 1,000** (68 fewer to 4 fewer) |
| **ICU-length** | | | | | | | | | | | |
| 2334 (10 RCTs) | serious ^a^ | not serious | not serious | not serious | none | ⨁⨁⨁◯ MODERATE | 1178 | 1156 | - | The mean iCU-length was **0** | MD **0.08 lower** (0.68 lower to 0.52 higher) |
| **Duration of mechnical ventilation** | | | | | | | | | | | |
| 1162 (5 RCTs) | serious ^b^ | serious ^b^ | not serious | not serious | none | ⨁⨁◯◯ LOW | 585 | 577 | - | The mean duration of mechnical ventilation was **0** | MD **0.58 lower** (1.47 lower to 0.31 higher) |
| **Adverse events** | | | | | | | | | | | |
| 2094 (11 RCTs) | serious ^b^ | serious ^b^ | not serious | not serious ^a^ | none | ⨁⨁◯◯ LOW | 120/1030 (11.7%) | 183/1064 (17.2%) | **RR 1.28** (0.87 to 1.90) | 117 per 1,000 | **33 more per 1,000** (15 fewer to 105 more) |

Figure S1 Risk of bias summary

**
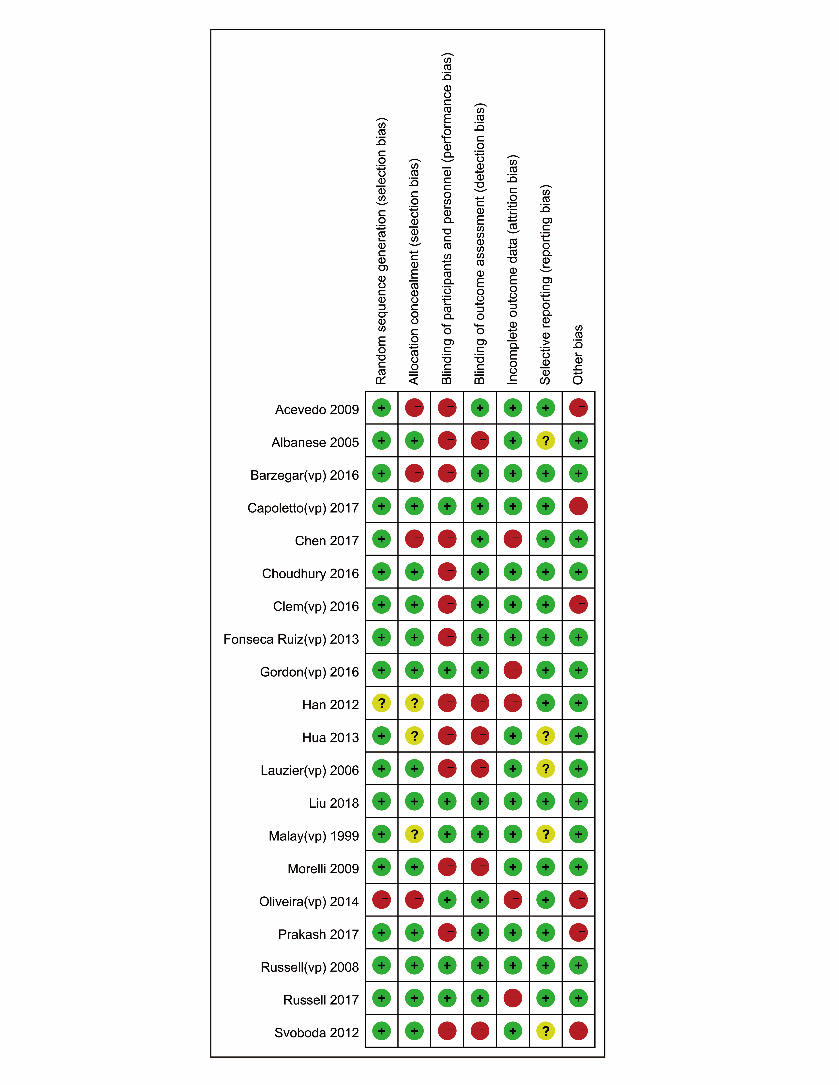
**

Figure S2 Risk of bias graph


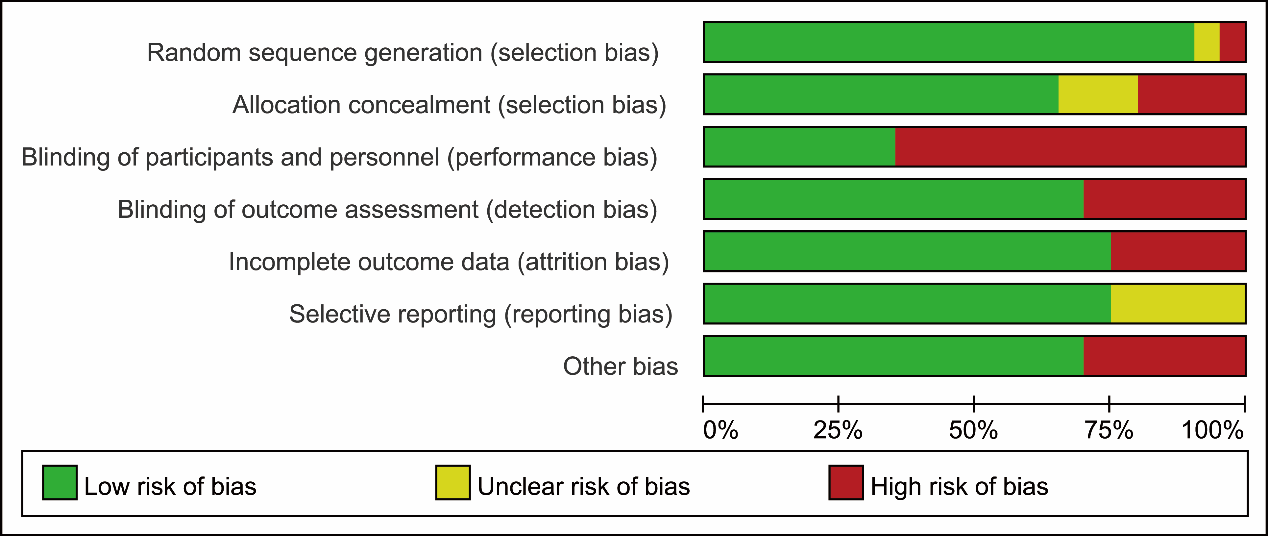


Figure S3 Forest plot for vasopressin or its analogues on cardiovascular events.


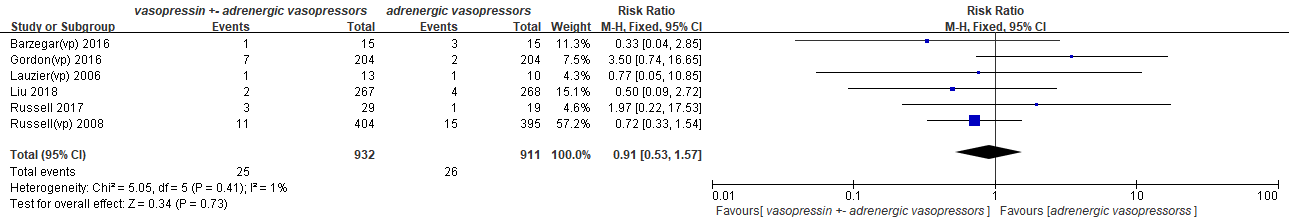


Figure S4 Forest plot for vasopressin or its analogues on arrhythmia


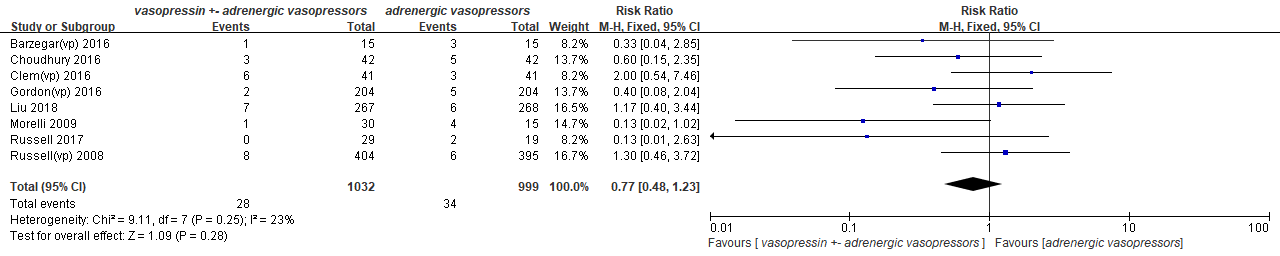


Figure S5 Forest plot for vasopressin or its analogues on mesenteric ischemia events

**
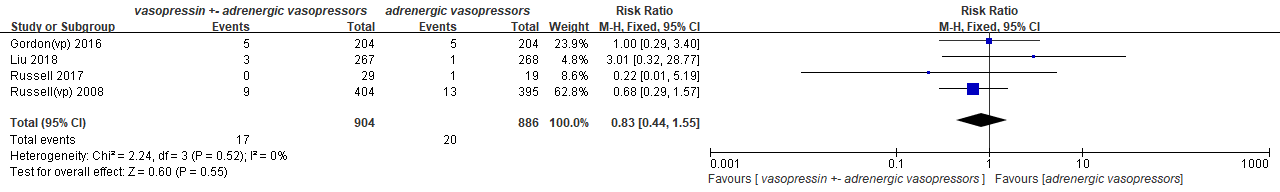
**

Figure S6 Forest plot for vasopressin or its analogues on diarrhea

**
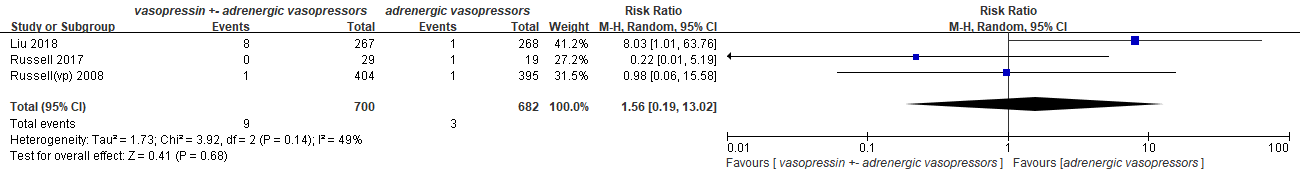
**

Figure S7 Forest plot for vasopressin or its analogues on cerebrovascular events

**
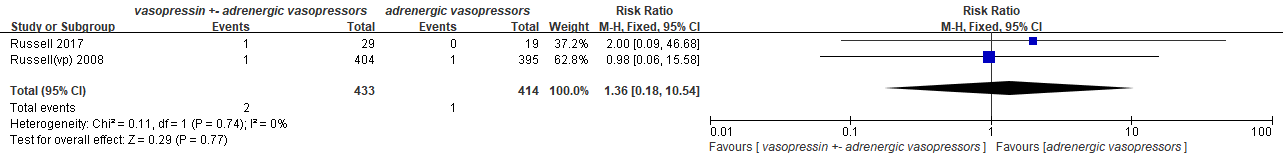
**

Figure S8 Forest plot for vasopressin or its analogues on hyponatremia

**
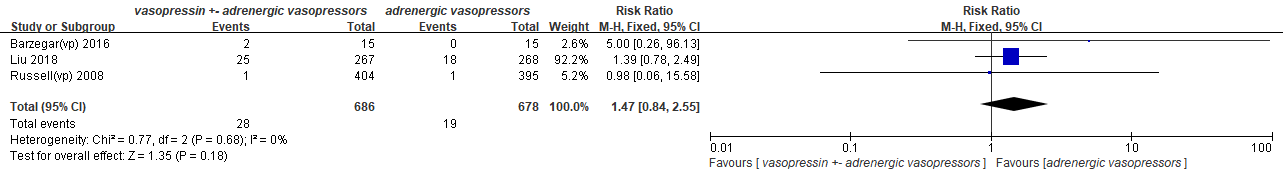
**

Figure S7 Funnel plot for publication bias

**
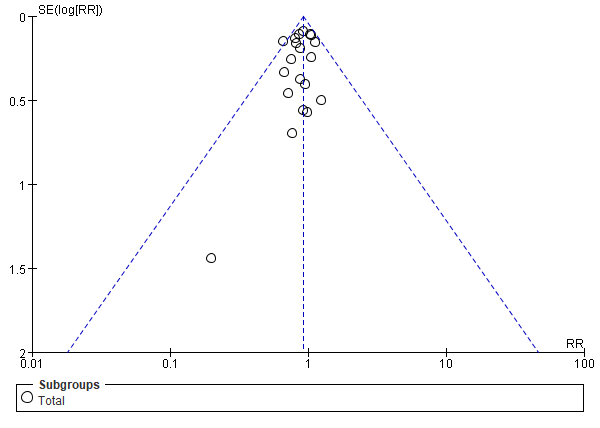
**
